# A hypothetical neurological association between dehumanization and human rights abuses

**DOI:** 10.1093/jlb/lsv015

**Published:** 2015-06-08

**Authors:** Gail B. Murrow, Richard Murrow

**Affiliations:** 1 Departments of Neurology and Neurosurgery, University of North Carolina at Chapel Hill, NC 27599-7025, USA

**Keywords:** dehumanization, empathy, hate speech, neuroscience, rights

## Abstract

Dehumanization is anecdotally and historically associated with reduced empathy for the pain of dehumanized individuals and groups and with psychological and legal denial of their human rights and extreme violence against them. We hypothesize that ‘empathy’ for the pain and suffering of dehumanized social groups is automatically reduced because, as the research we review suggests, an individual's neural mechanisms of pain empathy best respond to (or produce empathy for) the pain of people whom the individual automatically or implicitly associates with her or his own species. This theory has implications for the philosophical conception of ‘human’ and of ‘legal personhood’ in human rights jurisprudence. It further has implications for First Amendment free speech jurisprudence, including the doctrine of ‘corporate personhood’ and consideration of the potential harm caused by dehumanizing hate speech. We suggest that the new, social neuroscience of empathy provides evidence that both the vagaries of the legal definition or legal fiction of ‘personhood’ and hate speech that explicitly and implicitly dehumanizes may (in their respective capacities to artificially humanize or dehumanize) manipulate the neural mechanisms of pain empathy in ways that could pose more of a true threat to human rights and rights-based democracy than previously appreciated.

## INTRODUCTION

Tragically, history is replete with anecdotes suggesting that human beings have a tendency to dehumanize others and that this dehumanized perception is associated with reduced empathy for the pain of victims and with psychological and legal denial of their human rights and extreme violence against them. This is also consistent with political, legal, and ethical theory suggesting that an individual's humanity, personhood, or human dignity is essential to the acknowledgement and enforcement of that individual's rights.

We hypothesize that dehumanization is associated with the denial and violation of the human rights of victims, because it has an automatic dampening effect on the neural mechanisms of pain empathy that enable empathy for the pain and suffering of others. We hypothesize that, in an individual, who harbors implicit associations between a category of people and subhuman traits, such individual's neural mechanisms of pain empathy do not respond to the pain or suffering of that dehumanized category as robustly as to the suffering of other social categories more strongly implicitly associated with the human species and human traits. This may leave dehumanized groups, due to no fault of their own, unable to evoke the empathy needed to move other humans to act in accord with such groups’ rights.

We propose that this theory has implications for the philosophical conception of ‘human’ or of ‘legal personality’ in the philosophy and law of human rights. We also argue that it has timely bearing upon First Amendment free speech jurisprudence or on the doctrine of corporate personhood, as well as on consideration of the potential harm caused by dehumanizing hate speech. We propose that the new social neuroscience of empathy suggests that both the vagaries of the definition of ‘legal personality’ or legal fiction of ‘personhood’, *and hate speech*, that explicitly and implicitly dehumanizes may (in their respective ability to artificially humanize or dehumanize) manipulate the neural mechanisms of pain empathy in ways that pose more threat to human rights (international or as embodied in constitutional, rights-based democracies) than previously appreciated.

This paper's hypothesis is based on conclusions drawn from a review of empirical data from the neuroscience of empathy, dehumanization, and sociopathy, the psychology of prejudice, implicit bias, and dehumanization, and on psychiatric diagnostic criteria for diagnoses linked to disordered empathy and a lack of rights-based attitudes and behavior. It also relies upon previous, related insights from legal and ethical philosophy and theory.

In terms of the science cited, this paper offers only a brief review of existing data and does not present new, original, empirical data. Though we cite neuroscience and psychology in support of our thesis, this is a theory paper, which presents a hypothesis that is not scientifically proven.

The hypothesis is based upon the following, five, general, scientific findings, which will be elaborated upon in the body of this paper as follows.

1. Neuroscientific research suggests that pain, contrary to previous theories, is not merely an unpleasant sensory perception, but a homeostatic *emotion*, and that ‘empathy for “pain”’—which emerges from some of the same neural affective mechanisms active in pain—may supply emotional motivation for rights-based attitudes and behavior. This is consistent with previous theoretical models proposed in clinical psychiatry, as well as with more recent findings in neuropsychiatry.
2. The neuroscience of empathy or of ‘neural internal simulation’, including mirror neurons research, suggests that an individual's neural empathic (or neural internal simulation) mechanisms respond optimally to ‘conspecifics’, a biological term, which refers to members of the individual's own species. For example, an individual may ‘internally neurally simulate’, ‘mirror’, ‘feel’, or ‘empathize’ best with the actions, emotions, or pain of animals of the individual's own species.
3. Neural empathic mechanisms in humans appear to activate without *conscious awareness* or *control*, suggesting that the brain's identification of a conspecific, for purposes of activation of the neural mechanisms of empathy, may depend, not upon whom or what an individual *consciously believes* to be ‘human’, nor even upon whether the target^1^ of empathy is *biologically* human, but rather upon what the individual's non-conscious neural circuitry has been conditioned to most strongly, automatically, or ‘implicitly’ associate with the human species or human traits, physical and sociological.
4. Evidence of negative, relatively dehumanizing, ‘implicit associations’, also known as ‘implicit biases’, has been discovered in the psychological science of Implicit Cognition, which utilizes a research tool known as the Implicit Associations Test (IAT) to uncover unconscious associations, in the minds or brains of human subjects, between certain categories of humans and negative or less human-like traits.
5. The psychology of prejudice linked to linguistic factors, as well as discursive psychology, suggests that these implicit associations, between certain social categories and groups and the less than fully human, are conditioned and maintained by social discourse, including dehumanizing, demeaning, and defamatory hate speech or political propaganda.

The following is an outline of the evidence and argument in this paper.

Part I looks to historical evidence that reveals a strong correlation, if not a causal connection, between dehumanization and the denial of the dehumanized victims’ rights. It also briefly reviews the psychology of prejudice and dehumanization that developed in response to such historical events.
Part II explores the nature of neural internal simulation mechanisms, such as those involved in empathy for pain.
Part III looks at evidence and theory that pain and pain empathy are essential to rights-based attitudes and behavior and that the perceived humanness or personhood of rights claimants impacts both empathy for them and legal determination of their rights. Part III is divided into two sections.
  Section III.A. has two parts. Part 1 analyses the implications of the theory in this paper for current concepts of legal personality and corporate personhood. Part 2 explores the implications of this paper's thesis for hate speech jurisprudence in the USA.
  Section III.B. moves on to review scientific evidence that pain empathy may be essential to rights-based behavior, including evidence that pain is a homeostatic emotion with a behavioral, motivational component, as well as evidence from neuroscience and psychiatry which suggests that pain empathy, which involves the neural affective components of pain, underpins rights-based behavior.
Part IV reviews research suggesting that an individual's neural internal simulation mechanisms, such as those involved in pain empathy, respond more robustly to conspecifics, providing support for a hypothetical conclusion that the neural mechanisms of pain empathy, in a human subject, may not respond to the pain and suffering of human categories or groups who exhibit characteristics that have, instead, become implicitly associated, in that subject's underlying cognitive or conceptual system, with *non-human* animals and traits or with objects. This would be consistent with anecdotal observations.

## THE HISTORY AND PSYCHOLOGY OF PREJUDICE AND DEHUMANIZATION

Dehumanization is often a component of social prejudice, with some theories of prejudice proposing that the relative value others, persons or groups, or even non-human entities, is ultimately based upon their perceived degree of humanness, suggesting that all prejudice is based on assigning a greater or lesser degree of humanity to others.^2^ The interspecies theory of prejudice holds that prejudice is ultimately predicated upon the animal–human dichotomy.^3^ Research in anthropology and social psychology suggest that outgroups are universally distinguished as ‘not human’ or ‘not as human’ as ingroups.^4^

Further, of the myriad forms of social prejudice, dehumanization is historically associated with the most severe prejudice and with associated human rights violations and extreme violence.^5^ Such attitudes and behavior are also anecdotally associated with *reduced empathy* for the pain and suffering of dehumanized groups whose rights are infringed. In part III.B., we review diagnostic criteria and evidence from clinical psychiatry suggesting that disorders characterized by a such a lack of right-based attitudes and behavior involve disordered empathy or underlying differences in or impairment of its neural mechanisms.

Psychology's methodologies and tools for studying dehumanization and prejudice have evolved considerably over time. Gordon Allport, considered by many to be the founder of the psychology of social prejudice, studied it by recording and analysing historical evidence, as well as linguistic expressions, of prejudice and dehumanization.^6^

Allport's 1954 classic, *The Nature of Prejudice*, is still a frequently cited, highly respected reference, due not only to its insights, but because it was researched and written in the wake of World War II (WWII), and the Holocaust which was strongly associated with dehumanizing rhetoric and hate speech for which Allport coined the psychological term, ‘antilocutions’, which has been more recently described as follows:

*Antilocutions* (Allport, 1954/1979), from the Greek root meaning ‘against’ and the Latin root meaning ‘to speak’, are prejudiced speech, which include ethnophaulisms [ethnic slurs] as well as other linguistic factors in hostile prejudice, such as derogatory outgroup jokes.^7^

Allport's evidence was largely historical and anecdotal, versus empirical, though he focused novel attention on linguistic factors associated with prejudice, observing that prejudice could become embedded, maintained, communicated, and transmitted in *language*,^8^ and that ‘intense hostility is reflected in the antilocution of name-calling’.^9^

Hannah Arendt also theorized that dehumanization enabled the horrific, extensive human rights abuses of the Holocaust, further noting that there was something simple, commonplace, or ‘banal’ about such ‘evil’ or rights abuses in general and Nazism in particular, an unthinking, *automatic* submission to prevailing authority or law, regardless of the moral implications.^10^ She braved immense criticism by venturing to assert, based upon her observations at Adolf Eichmann's trial, that the historic crimes of Eichmann and lower level Nazi functionaries appeared to have some *automatic, unthinking* component.

In psychology, a behavior or attitude mediated by non-conscious processes in the mind or brain is referred to as ‘automatic’. Not only implicit associations, but the neural mechanisms of empathy, appear to operate automatically and non-consciously. Arendt's observations and philosophical conceptual analysis proposing that the Nazi functionaries’ historic rights abuses depended both on dehumanization and an *unthinking acceptance* of such rights abuses anecdotally supports our hypothesis that dehumanization automatically reduces the pain empathy that emotionally underpins rights-based attitudes and behavior.^11^

Researchers in dehumanized perception have more recently observed that such Nazi-like dehumanizing speech and attitudes have been associated, both long before and after Hitler, with extreme violence against victims: ‘The metaphor of Jews and Tutsis as cockroaches in Nazi Germany and war-torn Rwanda, respectively, Blacks as three fifths of a person in the US Constitution, and Iraqi prisoners portrayed as dogs by their American and British torturers are but a few instances’.^12^

Since the publication of Allport's classic work 60 years ago, methods and tools, such as the IAT and functional neuroimaging, have emerged for use in the study of social prejudice and dehumanization. Allport could only observe the *outward* expressions of prejudice and dehumanization, linguistic and behavioral, and had to rely on the affected individuals’ verbal description and conscious assessment of their state of mind and motivation or only upon the associations that they made *explicitly* between target social categories or groups and the less than fully human.

The psychological science of implicit cognition, employing the IAT, has allowed researchers, in recent decades, to look deeper into unconscious or ‘implicit’ associations that subjects harbor in their conceptual systems between more or less *ideally human* traits and target social categories or groups, such as the longstanding, stereotypical association of African-Americans with crime, or of women and girls with weakness and irrationality.^13^

Implicit associations have also been called, implicit stereotypes, implicit attitudes, or implicit biases, and are defined as associations between a social category or group and stereotypical or negatively valenced traits.^14^ The individuals who harbor such associations are not conscious of them and might likely reject them, if they were.^15^ However, such associations still unconsciously influence the individual's social decision-making and behavior.^16^ For example, psychological research suggests that many Americans have implicit forms of race-bias that unconsciously affect their social attitudes and behavior.^17^

The practical question is, ‘How are these dehumanizing implicit associations or prejudicial social hierarchies created and reinforced in the brain?’ This is a question that we are also addressing in another paper that we are currently working on. Very briefly, an influential theory, in discursive psychology, and which is consistent with Allport, is that these dehumanizing implicit associations or biases are conditioned discursively, or by discourse or language, such as hate speech, that makes similar associations between targeted groups and negatively valenced or non-human traits, animals, or objects.^18^

Current research suggests that dehumanization can also take far more subtle forms than the explicit or implicit association, direct or metaphorical, of a social category or group with non-human animals and objects, such as rats, pigs, trash, or bacteria. Dehumanization can manifest instead in the failure to *equally* associate targeted categories or groups with *innately human qualities*, such as mindedness, agency, or morality.^19^ This may be viewed more as ‘passive dehumanization’ and appears to implicitly associate *relative degrees* of *humanness* or of human qualities with different categories of humans, or to thus create implicit identity-prejudicial social hierarchies.

This is also supported by emerging research in cognitive *neuroscience*, utilizing functional neuroimaging (*f*MRI) to detect areas of the brain active in the performance of cognitive tasks. One such study was of men with implicit bias against sexualized women as determined by psychological IAT testing.^20^ When these subjects were shown images that sexualized women, the subjects had a reduced activation of the brain areas normally active in the attribution of agency, a human quality, to the women targets.^21^ Another neuroimaging study found that subjects’ contemplation of extreme outgroups, such as homeless people, who are stereotyped as threatening and disgusting, suggested that such outgroups were perceived by subjects as *less human*, or that such outgroups were thus dehumanized.^22^

Theory in the psychology of dehumanization proposes that it provides a means to *reduce empathy* toward the dehumanized person, category, or group for *self-defensive* or other *instrumental* purposes.^23^ This comports with anecdotal observations. A common, historical example is the dehumanization of a people or foreign group, who appear to pose an existential threat, whether this threat is real or merely perceived, eg based on paranoia, mass hysteria, vilifying rhetoric, demagogy, political propaganda, faulty intelligence, or other misinformation.

One group's dehumanization of another group, that poses a *legitimate* threat to the former, may reduce empathy for such an enemy's pain so as to enable extraordinary acts of aggressive self-defense by soldiers or so as to obtain political approval for a declaration of just war. Another example is the much studied dehumanization of patients (a.k.a. ‘cases’) by medical staff as a theoretical defensive strategy to balance or restrict the pain empathy that staff might feel for patients, and which empathy, while beneficial to both the staff and patients in many respects, may also, if extreme, negatively affect the medical staff's emotional health and thereby perhaps cloud their clinical judgment.^24^

More importantly, for our purposes, dehumanization also *maladaptively reduces empathy* for its victims and to no apparent end other than to facilitate social prejudice or to produce irrational fear, distain, or hostility. Demagogy is the intuitive art of rhetorically manipulating and reducing public empathy for the pain and suffering of people whom the demagog wishes to persecute for political reasons. Demagogs, such as Adolf Hitler, can often rhetorically project their own cruel intentions or inhumanity onto those they persecute so as to make their own rights violations or crimes against humanity appear morally justified or necessary for the public defense. This projection of one's own inhumane intent onto others can be a conscious calculated strategy or an automatic, unconscious, psychological defense mechanism that thus appears related to *implicit* association. This ‘subconscious’ mechanism is known, in psychoanalytical psychiatry, as, ‘projective identification’.^25^

The oppression, enslavement, and abuse of women and racial or ethnic minorities over time has likewise often been justified by claims that these oppressed classes lack the *human capacity* to care for themselves or to feel human pain and suffering, a claim that suggests that their oppressors lacked pain empathy for them.^26^ According to our theory, such a lack of pain empathy enables oppressors to exploit other humans by avoiding the deterrent emotional discomfort of pain empathy, also known as ‘*pangs* of sympathy’, which would otherwise send an emotive signal that such behavior is unethical, immoral, or an infringement of basic human rights.

However, the underlying, *neuroscientific* question is: How could dehumanization reduce empathy, not simply in the conscious mind, but in the *non-conscious brain?* How could dehumanizing implicit associations function to *modulate or dampen the activation of the neural mechanisms of pain empathy* in responding to a dehumanized social target?

## THE NEUROSCIENCE OF EMPATHY

We cannot, in the scope of this paper, review the neuroscience of empathy other than in limited, simplified terms that cite a small amount of the available, empirical data. An influential model of empathy is the Perception-Action Model (PAM) proposed by Preston and de Waal. It is a unified model of empathy that integrates theoretical and philosophical views of empathy with empirical findings from different scientific fields and relies upon research in both humans and animals. For a detailed description of PAM, see Preston & de Waal (2002).^27^

As we noted above, there are a confusing number of concepts of and terms for empathy, such as ‘sympathy’ and ‘compassion’. Preston and de Waal have proposed that ‘empathy’ should be more accurately viewed as a *broad phenomenon* encompassing many processes that depend upon PAM and result in what are variously termed, empathy, sympathy, emotional contagion, cognitive empathy, guilt, altruism, and identification.^28^

They posit that this broad phenomenon has a *neurophysiological* basis described as an *emotional linkage* between conspecifics that arises when the perception, by one individual, of another's behavioral state, automatically activates neural representations of that same state in the observer's brain.^29^

For example, if one individual observes another conspecific grasping an object or experiencing pain, there is an automatic activation, in the passive observer, of some of the same neural structures that would be active, if the observer were grasping the object or experiencing the pain firsthand. Such findings in neuroscience have led to the general theory, elaborated in PAM, that it is by this non-conscious ‘neural simulation’ of others’ actions or emotions that one conspecific ‘empathizes’ with another or automatically understands the other's behavior or internal states. This kind of ‘empathy’ is ‘embodied’ or *neurophysiological*, meaning it occurs in the unconscious brain and body, versus conscious mind, though it may coincide with or precede conscious, empathic thoughts.

In addition to PAM, this theory has been known as simulation theory, the theory of embodied empathy, the shared (neural) networks hypothesis, and the shared (neural) representations hypothesis. It has also been theorized to provide a neural mechanism for forming what psychologists and philosophers refer to as a ‘theory of mind’ or a sense of others’ underlying thoughts, feelings and goals, which is vital to a social species.

The exact parts of the human brain or neural components that mediate this neural ‘simulation’ or ‘mirroring’ of conspecifics’ actions and emotions, have not been clearly determined, but have been referred to, in specific and general terms, as: mirror neurons; specialized spindle cells called Von Economo neurons; the mirror neuron system; human mirror system; and neural internal simulation mechanisms. Below we review research on mirror neurons in human and non-human primates using single-neuron recordings, as well as research on pain empathy in humans using functional imaging of macroscopic neural structures. However, we emphasize, in accord with PAM, that this embodied ‘empathy’, generally referred to as neural internal simulation or the human mirror system, likely involves interconnected neural components and circuits neuroscience has yet to fully describe.

In the 1990's, neuroscientists in Italy discovered a special kind of neuron hailed, the ‘mirror neuron’, because it appeared to facilitate an automatic mirror neural simulation in one individual of certain motor acts they observed another individual performing.^30^ This simulation or ‘mirroring’ was theorized to provide an automatic sense or understanding of the intentions or goals of the actor, in the observer, and thereby to supply a neural mechanism for attributing states or feelings to others at the *cellular* level.

Mirror neurons were discovered in rhesus macaque monkeys in the ‘F5’ region of the ventral pre-motor cortex and inferior parietal cortex, an area theorized to be a homolog to Broca's area in humans, which is integral to language.^31^ These neurons were discovered using single-neuron microelectrode recording, which revealed that such neurons were activated both when the monkey observed another individual or conspecific perform a motor act and when the monkey performed the act itself.^32^ This created what was called an ‘off-line’ *mirror neural simulation* of the observed action in the observer.^33^

Although it has been widely assumed that mirror neurons exist in humans, there is currently very little direct evidence that they do, because the invasive single-neuron microelectrode recordings that were used to discover them in non-human primates are not generally considered to be ethical for use in experimentation involving human subjects. While such recordings are performed in humans for diagnostic and therapeutic purposes, for example to assist neurosurgeons in targeting neural structures, recordings done for *purely experimental* purposes could unnecessarily expose the subjects to the risks associated with open surgery on the brain, such as bleeding, seizures, infection, death, or brain damage. Animal rights proponents might argue that such experimental recordings in non-human animals should not be allowed for the same reasons. However, US law does currently allow such recordings in a variety of non-human species, including rats, monkeys, and ferrets.

In humans, less invasive techniques than single-neuron recording—including *f*MRI, positron emission tomography, magnetoencephalography, electroencephalography, and transcranial magnetic stimulation—have yielded evidence that a similar mirroring system does exist in the human brain, whether in the mechanism of the mirror neuron or via other neural mechanisms in areas implicated by such research,^34^ including the pre-motor cortex, parietal lobe, caudal area of the inferior frontal gyrus, anterior mesial frontal cortex, insula,^35^ and anterior cingulate cortex (ACC).^36^

While the bulk of the above research suggested that neural internal simulation mechanisms simulated the *motoric* acts of other individuals, later research provided evidence that such embodied simulation may also be the neural platform of *empathy for pain*.^37^ Though much neuroscientific research has looked at pain empathy, the ground-breaking study was an *f*MRI study conducted by Singer et al., in 2004, which provided early evidence that when one individual observed a ‘sign’ that another individual was in pain, some of the same neural components that are active in the firsthand experience of pain were also active in observing or imagining another person in pain, but only the neural *affective* or *emotional* components of pain, not the *physical* components.^38^ The areas activated were the bilateral anterior insula (AI) and dorsal ACC.^39^

These researchers go on to describe this in practical terms or to explain how such empirical findings comport with dominant theoretical accounts of empathy:

The key suggestion is that observation or imagination of another person in a particular emotional state automatically activates a representation of that state in the observer, with its associated autonomic and somatic responses (‘automatic’ refers to a process that does not require conscious and effortful processing but can nevertheless be inhibited or controlled). The philosopher Susanne Langer has described it as an involuntary breach of individual separateness.^40^

In addition to such *f*MRI studies of pain empathy, there is other neuroscientific evidence that suggests mirror neural simulation plays a role in pain empathy. Some of the extremely limited number of single-neuron recordings that have been done in humans to investigate whether mirror neurons exist in human primates do provide some direct evidence that they do,^41^ and some of these recordings were performed specifically to investigate *pain empathy*.^42^ Hutchison et al., during the course of cingulotomy surgery for refractory conditions, recorded from single neurons in the ACC, the same area of the cingulate that was found to be active in pain empathy as indicated by *f*MRI in the above study by Singer and team.^43^ Hutchison's group was able to isolate several neurons in three surgical subjects that activated both on the application of a painful stimulus and the observation of a painful stimulus applied to another human.^44^ This suggested mirror neurons in the ACC that could be a neural mechanism of pain empathy.

A later *f*MRI study conducted by Morrison and Downing to investigate the neural correlates of both felt and seen pain, found, in six of 11 subjects, a small area of the ACC and AI where felt and observed pain overlapped, and which therefore appeared to support mirroring as a basis of pain empathy.^45^ However, there was no such overlap in the other five subjects.^46^

However, there is not nearly enough of such data at this time to confirm either that mirror neurons exist or do not exist in the human ACC or that they are essential to pain empathy. Convincing evidence is not likely to be available for some time, as the best evidence, according to an ‘evidence-based medicine’ standard, would require larger validated cohort studies employing single­-neuron recordings, and current recording techniques, as indicated above, place subjects at too much risk to perform such recordings for purely experimental purposes. The Hutchison study was likely approved because the subjects were undergoing rare, therapeutic, surgical removal of the brain areas that were recorded from, so that the recordings did not significantly increase risk. Finally, though we suspect that mirror neurons may play a role in pain empathy, we do not propose that they provide the sole neural mechanism of empathy.^47^

We are proceeding, in this paper, on a general theory—akin to the ‘PAM’ of empathy shared by a number of scientists and which is based in part on the ‘Perception Action Hypothesis’ that arose from mirror neuron research—that what we have referred to here as, ‘neural simulation mechanisms’, whether in the form of mirror neurons or other neural mechanisms, do exist in the human brain, and that such neural simulation is the source of the pain empathy theorized to motivate prosocial and helping behavior.^48^ In the next section, we will move on to examine evidence and theory that it is pain empathy that provides emotional motivation for rights-based and prosocial behavior.

## RELATION OF PAIN EMPATHY TO RIGHTS-BASED VS. ANTISOCIAL CONDUCT

### Arguments From and Impact Upon Jurisprudence

Legal philosophers, including Cesare Beccaria and Jeremy Bentham, have long proposed that pain and empathy (‘sympathy’)—which have been viewed as related but different forms of natural discomfort—are essential to motivating compliance with the moral dimensions of the law, including criminal law and equality-based rights, or to providing a natural, utilitarian motivation to accept and act in accord with others’ basic human rights.^49^

Punishments for violation of such law and rights have reflected this theory that direct, personal pain, physical and emotional, motivates moral, prosocial behavior. For example, both positive law and religious law threaten physically or emotionally painful punishment for violations of the law *ex post facto*, or in jail or the afterlife. The more indirect, vicarious pain of empathy, conversely, was thought to prevent or deter crime *before the fact*. Bentham described these pre-emptively, punitive ‘pains’ as, ‘The pains resulting from the view of any pains supposed to be endured by other beings. These may also be called the pains of good will, of sympathy, or the pains of benevolent or social affections’.^50^ In political and moral theory, such empathy may have been referred to as the ‘sense of *equality*’, which was also theorized to contribute to rights-based behavior.

The term ‘“human” *rights*’ is prescriptive as well as descriptive, for entitlement to such rights has long been based, in legal, political, and moral theory, upon the subject claimant's humanness or personhood. However, as history and past legal decisions^51^ indicate, this is ‘humanity’ or ‘personhood’ as determined by the social, moral, or legal decision maker, or reigning discourse, rather than by *biology*.

This supports the hypothesis in this paper that it is not whether an individual or social group is *human*, but whether they are perceived or portrayed as such, explicitly or *implicitly*, that more reliably predicts whether the group will evoke the pain empathy in conspecifics necessary to emotionally motivate the latter to acknowledge and behave in accord with that group's human, civil, Constitutional, or equality-based rights.

Again, the critical, practical question is, ‘How are these emotionally misleading implicit associations, between a targeted social group and the less than human, formed in society and in the brain?’ One theory, discussed above, and which seems the most obvious, is that such *implicit* associations or biases are conditioned and sustained by social discourse, such as hate speech, that makes similar *explicit* associations between targeted social groups and non-human life forms, such as pigs and rats, or between people and objects, such as dirt and trash.^52^ We will further analyse these proposed effects of hate speech on empathy later in this section.

#### Implications for legal personality

However, first allow us to examine a perhaps less obvious way that the language used to define what is ‘human’ or a ‘person’, in relevant *law* and *ethical theory itself*, may enable dehumanization and distorted interpretations of equality-based rights. Sophie Oliver argues that both the law's and ethical theory's persistence in defining humanity in abstract, amorphous, metaphysical terms, such as, ‘dignity’, or its abstract components, including rationality, mindedness, or moral agency, still allows certain social categories or groups, that are arbitrarily viewed as lacking these abstract elements of human dignity, to fail to be recognized as fully human and fail to have their human rights recognized.^53^ A historical example is that of how women were long denied equal rights because of their alleged *irrational, emotional, unjust*, or *non-impartial* nature. Oliver proposes remedying the problem by viewing ‘dignity’ as an ‘embodied’ human quality that refers to the ‘corporeal’ nature and experience of human beings, particularly their pain and suffering.^54^

We would go further to suggest not simply that dignity be viewed as an embodied human quality, but that the human body, in general, in its live, post-birth,^55^ biological form, be viewed as the entity that is entitled to human rights, regardless of the abstract qualities that may or may not be attributed to it, such as dignity, morality, or personhood, and which can have different definitions across time, culture, and legal systems. Though such a proposal is not new, there may be new support for it, if the empathy on which human rights depend also depends on the rights holder being implicitly viewed as *human*.

We acknowledge that this simple species-based definition of human rights holders does not address or resolve the harder current and futuristic cases wherein the question is when, or if, a putative rights holder is fully human or sufficiently human-like to be entitled to legal rights or protection, whether those cases involve a human fetus at various stages of gestation or future advanced forms of artificial life or intelligence. Future work with hybrids, chimeras, or clones may also pose ethical challenges.

However, if the language of the law of human rights and ethical theory were, as Oliver suggests, to better emphasize the ‘embodied’ nature of ‘humanness’, as opposed to its more abstract qualities or descriptions, such as ‘dignity’ or ‘personhood’, perhaps this could help avoid any further denial of rights to groups that are *indisputably human* but that are still prejudicially, *implicitly* viewed by some sectors of the public as *other*, or *less than*, human. For example, in the past, indigenous peoples were often denied human rights because they were viewed, by colonists and explorers, as ‘savages’ or ‘animals’ rather than as ‘dignified’ ‘persons’. This phenomenon continues today in many forms, for instance, in the arbitrary denial of basic rights to lesbian, gay, bisexual, and transsexual (LGBT) people, because some cultures or religious groups decline to view LGBT human beings as ‘moral’ or ‘normal’ *humans*.

Perhaps, the use in human rights law and ethical theory of such abstract, slippery, evasive terms as ‘dignity’ and ‘personhood’, to describe who or what is *human* or *Homo sapiens sapiens*, unintentionally, unwittingly retains, in rights jurisprudence, those same terms that have, throughout history and into the present, given some groups wiggle room to psychologically deny that other groups are fully human and to deny such others’ rights.

The science and theory in this paper suggest that such implicit dehumanization or implicit social hierarchies further reduce the *pain empathy* for these dehumanized groups that emotionally motivates rights-based behavior toward them. If the pain empathy, that may be the natural enforcement mechanism of human rights, depends upon legal subjects viewing rights holders as members of the human species, then why shouldn't the positive law make it more explicit that membership in the ‘human species’ constitutes sufficient evidence or an English law type irrebuttable presumption of entitlement to human rights?

Perhaps the law could thus set a more firm example or send a clear, unequivocal message that the rule of law does not (and nor should its subjects) view social hierarchies, individual identifying features (physical, sexual, religious, and sociological), or culturally variable views of dignity or morality, as grounds for denying any ‘human’ being her or his ‘human’ rights.

There are other ways in which we suggest that the notion of legal personhood may have an unintended unconsciously negative impact upon human rights. The legal term of art, *legal personality*, has long been accepted in the philosophy of law and has provided a convenient way by which to extend necessary, legal privileges not just to ‘natural persons’, ie humans, but to fictional or ‘juridical persons’, such as corporations and groups, including the right to enter into and enforce contracts or to sue and be sued. However, we argue that this fictional personification of entities and animals other than humans, while convenient, may unconsciously manipulate empathy in ways that harm or undermine the equality-based rights of *human* individuals, social categories, and groups.

First, we propose that the well-intentioned use of this strategy to, for example, artificially personify or humanize non-human species (eg by labeling non-human primates ‘persons’ to promote their humane treatment) may be just as harmful to *human* rights as using it to *dehumanize*. Because if conceptions of ‘human’ are discursively constructed by language, linguistic practices, or social or legal discourse, whether hate speech or the language of the law, then the fictional or metaphorical equation of human beings with non-human animals may not only automatically, unconsciously condition *implicit associations* between lab chimpanzees and humans, but reciprocally *reinforce* old underlying prejudicial implicit associations between, for example, apes and German Jews or African Americans, or between women and objects, property, or breeding stock.

If forms of life or entities, *such as* non-human animals used in lab experiments, are also thought to be entitled to rights akin to those of an individual human, even if the putative rights-holders are a human collective or a human fetus, let us not use, as a *rationale* for extending rights to such life or entities, their mere metaphorical or verbal association with a human body or their fictional legal designation as a ‘person’, which cognitive linguistics suggest may automatically, metaphorically map unintended qualities of the source domain (the human body) onto its target domain (embryos or lab mice).^56^

For example, as the demagog can attest, such analogical mappings can mislead as well as elucidate. Associating rats with people might not only empathically, implicitly map the human-like capacity for pain onto lab rats, but bidirectionally map the qualities of rats, also viewed as stealth disease carriers, onto target social groups labeled as ‘rats’ in hate speech, perhaps causing such groups to evoke, like rats, emotions of fear and disgust, instead of empathy.

We propose that this legal strategy of naming any life form or entity, a ‘person’, to which one wishes to grant rights or legal protection is not simply due to previous legal precedent or language, such as the use of the words, ‘any person’, in the Equal Protection and Due Process Clauses. Instead, we propose that this strategy also intuits this paper's theory, that it is whether an entity is implicitly associated with the *human species* that ultimately, empathically moves humans to act toward in it ways that promote or enhance its survival or to treat it in a rights-based manner. The demagog's strategy of, by contrast, dehumanizing or denying the personhood of those groups whose rights he wishes to violate for personal or political purposes, is likely based on the same intuition.

In fact, we further suspect that an individual social, moral, or legal decision-maker's initial, gut feeling about whether a given entity deserves rights is predetermined by whether the entity is implicitly associated, in the individual's brain, with human traits. This is not a *conscious* form of social prejudice or something over which the individual has conscious knowledge or control, but an innocent result of sociocultural conditioning. Research suggests that even the most socially liberal harbor unconscious, implicit biases.

Returning to our argument that, legally, metaphorically, or linguistically equating non-human species with ‘persons’ could implicitly dehumanize certain social categories, particularly the historically oppressed or dehumanized, we turn to human rights case law. The European Court of Human Rights (ECHR), in its judgment on the merits, in *PETA Deutschland v. Germany*,^57^ upheld an injunction by the German Courts that prevented animal rights organization, PETA (People for the Ethical Treatment of Animals), from running ads comparing mass farmed livestock to German Jews in concentration camps. The ECHR held that the injunction did not violate PETA's right to freedom of expression, under Article 10 of the European Convention on Human Rights, because, consistent with the German Courts’ rulings, the ads violated the *personality rights* of affected German Jews.

The injunction was first sought, in German courts, by German Jews who had survived the Holocaust as children. The Berlin Regional Court granted the injunction based on a finding that the ads violated the *human dignity* and *personality rights* of the plaintiffs. Upon appeal by PETA, the Federal Constitutional Court of Germany upheld the injunction on the grounds that it did violate the affected German Jews’ ‘personality rights’, though the Court had doubts about whether it also violated their human dignity.

Note that ‘personality rights’, in civil law jurisdictions, are more of a *property*, than a *personal*, right. They guarantee the right to own one's personal image or the right to protect or control it or one's reputation or persona from base, commercial exploitation. Personality rights are usually inheritable, so that one can also protect the image, memory, or reputation of deceased relatives from thus being exploited or demeaned. One plaintiff in this case had lost her family in the Holocaust.

The ECHR found that the German Courts’ injunction had a ‘legitimate aim of protecting the plaintiffs’ personality rights and thus “the reputation or rights of others”’.^58^ Both the holdings of the ECHR and the German Courts suggest a sense, consistent with the theory in this paper, that portrayal of humans, particularly a historically oppressed category, in this manner, or as virtually equal to a lower species, might not only increase empathy for the suffering of the animals thus personified, but unintentionally reciprocally hurt and reduce empathy for or banalize the suffering of Holocaust victims by implicitly reconstructing them, in Nazi terms, as ‘animals’. The ECHR's wording also suggests that equating animals with a certain social group not only affects the reputation or rights of the individuals specifically targeted, but those of *others* in the same social category.

All Courts involved found that PETA did not *intend* to demean or trivialize the suffering of Holocaust victims or survivors. However, such effects may, particularly in their downstream social and cultural manifestations, be thus unforseen, for they may result from the underlying interaction of two, *non-conscious* neural mechanisms, those of implicit semantic associative learning *and* pain empathy.

Therefore, while the authors of this paper have deep sympathy for animals kept in cruel conditions, as, for instance, in battery farming operations, if there are other ways to advocate for better treatment of such animals (as the Courts found in the *PETA* case) than depicting or referring to them as people, especially as a historically oppressed, persecuted people, then such other means may better protect ‘human’, as well as ‘animal’, ‘rights’.

Having discussed how we propose that granting legal personhood to *non-human species* may reduce empathy for, and thus threaten the rights of, humans by creating or maintaining implicit associations between dehumanized peoples and non-human animals, we will now move on to consider whether attributing legal personhood to *corporations* could have a similar effect upon the empathy for, or rights of, natural persons or humans.

The legal concept of ‘corporate persons’ has been a useful, largely accepted, legal fiction as used to extend, to corporations, legal privileges necessary to conduct business operations. However, its more recent use, by the US Supreme Court, in *Citizens United v. F.E.C.*, to extend to ‘corporate persons’ the individual right to unfettered free speech in political campaigns and discourse, has been more controversial, due to its practical effect in allowing unlimited spending from corporate treasuries on electioneering.^59^

Justice Stevens, in his dissent in *Citizens*, wrote, ‘[The Framers] had little trouble distinguishing corporations from human beings, and when they constitutionalized the right to free speech in the First Amendment, it was the free speech of individual Americans that they had in mind’.^60^ He argues that the majority's refusal to distinguish corporate from natural persons in this case may ‘give corporations “unfai[r]’ influence” in the electoral process’,^61^ ‘[marginalize] the opinions of real people’,^62^ ‘promote corporate power at the cost of the individual and collective self-expression the [First] Amendment was meant to serve’,^63^ and threaten the people's faith in the democratic political process, and their assurance that it will not be skewed or corrupted by the undue influence of large corporate war chests on candidates, legislators, or public officials.^64^

We theorized earlier that the legal strategy of asserting that *non-human species* are ‘persons’ is used not just to bring other species into the protection of laws that reference and protect ‘persons’, but that it is also used intuitively to create the *empathy* for other species that is needed to emotionally motivate people to extend animals’ rights. We posit that the legal fiction or rhetorical device of the *corporate person* likewise works, not only to legally argue that corporations are entitled to human rights, but to manipulate empathic emotion so as to make it *feel, emotionally*, as if corporations *are*, in fact, ‘persons’ and thus entitled to human rights—whether this is true as a matter of fact, logic, law, or ethics.

While the personification of corporations may implicitly *objectify* natural persons, it does not seem to us as likely, as the personification of non-human animals, to implicitly associate humans with lower animals or life forms, as corporations are not living entities.

However, if the practical result of viewing a corporation as a ‘person’ under the law can, in some cases, as Justice Stevens argues in *Citizens*, still undermine the rights of *natural persons*, then perhaps calling corporations, ‘persons’, can, like calling monkeys, ‘persons’, also have an adverse effect on *human* rights, but, this time, not by the implicit association of humans with non-human species or capabilities, but instead by the implicit association of the non-human corporate form with distinctly human biological capabilities.

For, as Justice Stephens argued, ‘corporations have no consciences, no beliefs, no feelings, no thoughts, no desires’^65^ which are in fact the embodied human qualities that the individual, human right to freedom of speech and expression was intended to protect.

#### Implications for hate speech

Returning to hate speech and relevant US Supreme Court precedent, broad protection has long been given to hate speech, particularly with a political purpose, unless it is interpreted to provide incitement to immanent lawless action or constitutes a true threat. In *Brandenburg v. Ohio*, the Supreme Court held, ‘Freedoms of speech and press do not permit a State to forbid advocacy of the use of force or of law violation except where such advocacy is directed to inciting or producing imminent lawless action and is likely to incite or produce such action’.^66^ In this way, the US legal system gives greater legal protection to hate speech than many other countries, including the UK, Israel, Canada, Australia, and a host of European nations, all of which impose moderate legal restrictions on hate speech.

While the American system has provided exceptionally strong protection to hate speech, some have argued that First Amendment rights need to be better balanced against the rights of people targeted by such speech to equal protection under the 14th Amendment, or that the right to speak hate must be balanced against the interests of the targets in *social equality*.^67^ Other scholars propose that hate speech is threatening to the *personhood, human dignity*, or *social equality* of those social categories and groups that it targets.^68^

This second view is more consistent with the theory in this paper. To explain, let us translate the legal fiction of ‘personhood’, as well as ethical theory's ‘human dignity’, into the question of whether a rights claimant is implicitly viewed as ‘human’. Next, let us translate liberal political theory's ‘“sense” of equality’ into its physiological correlates, relabeling it as, the ‘social emotion’ or sense of ‘empathy’. Now that we are speaking the same language, if hate speech conditions, in speakers and listeners alike, an implicitly dehumanized view of its human targets, and, if this reduces the empathy for targets that motivates prosocial behavior toward them, then, *in legal terms*, it also reduces the *sense of equality* that conspecifics have toward the targets and thus deprives the latter of equal protection of their rights, which is provided not simply by positive law, but by the capacity to evoke *empathy*. Therefore, if hate speech does condition or maintain dehumanized perception of those groups it targets, then it may pose a true threat to such targeted groups’ safety or human rights.

Robert Mark Simpson recently suggested that hate speech might be justifiably legally restricted when there are ‘good reasons to think that hate speech contributes to, and/or bears responsibility for, the establishment and perpetuation of identity-prejudicial social hierarchies, and the harms and disadvantages that individuals experience as a consequence of those hierarchies’.^69^

We do not claim that the evidence and theory presented in this paper is sufficient to justify general legal restrictions on hate speech under Simpson's test. We simply offer a neuroscience-based theory regarding how hate speech might be threatening to its targets in ways of which jurists, scholars, and scientists have not perhaps been previously aware.

Further, there seems to be growing tension, in America, between liberal political theory's dual commitments to free speech and social equality. This tension is apparent in increased calls for more legal restriction of hate speech, particularly on the Internet. People and political leaders are alarmed not only by how rapidly hate speech can spread online, but how fast it can incite horrific, widespread acts of terror and violence, whether perpetrated by lone wolves or in a coordinated fashion. The most compelling example is the rapid rise and current deadly threat of ISIL or the Islamic State of Iraq and the Levant.

Democratic Senator, Ed Markey, has introduced a bill that calls for governmental investigation of hate speech on the Internet (S. 2219),^70^ while Democratic Representative, Hakeem Jeffries, has introduced a similar bill (HR 3878).^71^ Legal commentator, Tiffany Kamasara, has argued that, ‘New standards are needed to address the growing plague of Internet speech that plants the seeds of hatred, by combining information and incitement that ultimately enables others to commit violence’.^72^

The hypothesis in this paper may shed light not just on how hate speech might thus *enable* or *incite* violence, but how it can do so in such an apparently unthinking, ‘banal’, perfunctory, *rapid* manner. This may be because of the ‘automaticity’ or the automatic, *unconscious* effect of hate speech on the neural mechanisms of pain empathy and on empathy for the human targets.

While sudden, violent surges of hatred seem to make no sense, that may be because, under the theory we present, hate speech goes *around* the *conscious mind* to directly attack the *emotional* mechanisms of empathy or moral restraint. This proposed automatic effect of hate speech on the brain, coupled with the ability of such a verbally transmitted, emotional contagion of disempathic hatred to be spread at exponential speed over the Internet, or this capacity of hate to go viral in the violent flash of a mob, may warrant a reconsideration of current, jurisprudential concepts of ‘immanence’ and ‘incitement’, as has long been argued by other commentators.^73^

In his famously eloquent defense of free speech in *Whitney v. California*, in 1927, Justice Brandeis urged, with regard to immanence, ‘If there be time to expose through discussion the falsehood and fallacies, to avert the evil by the processes of education, the remedy to be applied is more speech, not enforced silence’.^74^ However, he could not then foresee a literally revolutionary Internet age in which hate speech and misinformation can spread so far and fast, that there may, indeed, *often not be sufficient time* to discuss and expose falsehoods or to avert the evil acts they incite. He also could not foresee an age in which the form of *education* most relied upon globally—especially by impressionable young people who may spend more time online than in class or with parents—would be a Worldwide Web of too often unexamined, unchallenged, unfiltered speech, the sheer volume or information overload of which might do more to *obscure*, than *reveal*, truth.

It is not just the obvious forms of hate speech that use this rhetorical method of dehumanizing the ‘other’. *Political* rhetoric and discourse in the USA today seems to be plagued by a camouflaged, passively dehumanizing form of name calling and incivility which appear to be automatically mirrored in a highly divided and polarized society.^75^ Political issues are debated not only by political leaders and candidates, but by a cacophony of affiliated, theatrical, talking heads, comics, and talk show hosts who often cater to one side of the political aisle by making fierce fun of the other, by derogatory political outgroup jokes, or, in other words, once again by the same kind of political *name calling* that aims to associate, in the brains of listeners, the political views of the other side with such ‘others’ *ignorance, stupidity, prejudice, inhumanity, selfishness, cruelty, Godlessness*, or *unfeeling or sinful nature*, each side having different names for this same alleged ‘inhumanity’.

Some such dehumanizing speech may be obvious or explicit, such as referring to another politician as animal feces, or it may be more implicit or ‘passive’ and seek to dehumanize its targets by implying that they are less endowed with defining human qualities, such as intelligence, dignity, morality, compassion, sympathy, or empathy. This is ironic, as it seems that the more or less subconscious intent and purpose of such rhetoric is to deprogram public empathy for its targets.

There also appears to be similar reliance on dehumanizing and vilifying rhetoric and hate speech in those strains of political and militant Islam that drum up the religion-based prejudice that appears to be linked to an ensuing lack of empathy that enables acts of terrorism by ordinary individuals who otherwise appear extraordinarily devoted to conforming with what they view as religious interpretations of goodness and morality.^76^

Historically, such rhetoric has been a dangerously divisive, even deadly, political tool. Perhaps, today, political and spiritual leaders of every ideology, as well as social and environmental activists, should avoid deploying such language. The temptation is great, for there is an apt intuitive sense, as well as polling data and election results, to suggest that such rhetoric and incivility have a natural power to sway public opinion, empathy, or elections by inciting class, race, gender, religious, or interparty warfare. Yet, our theory suggests that this same rhetoric may actually block real, political progress, compromise, or willingness to see the other side's genuine concerns, because one side can no longer even see the other as sufficiently ‘human’ to *evoke empathic concern*.

Official, political use of such linguistic tactics to manipulate public empathy might also set an official example that name calling, which implicitly dehumanizes or demeans opposing political groups, is ethically acceptable. Sarah Sorial recently argued, ‘The danger in mischaracterizing an instance of hate speech by calling it academic or political debate is that it risks normalizing the views and sentiments that are expressed and accepting those views as an important part of our political and academic discourses’.^77^ Could this ‘name calling’, that actively or passively *dehumanizes*, ever have a political purpose other than to win political elections or debates by going around the facts to incite hatred or prejudice against people or candidates with opposing views?

Czeslaw Milosz, late WWII poet and Nobel laureate, who also studied law in his youth, left behind a body of work that explores how the rhetoric of totalitarianism and fascism played a role in the catastrophic human rights abuses of that dark period. His observations seem to have hauntingly current relevance:

European culture entered a phase where the neat criteria of good and evil, of truth and falsity, disappeared; at the same time, man became a plaything of powerful collective movements expert in reversing values, so that from one day to the next black would become white, a crime a praiseworthy deed, and an obvious lie an obligatory dogma. Moreover, language was appropriated by the people in power, who monopolized the mass media and were able to change the meaning of words to suit themselves.^78^

Milosz refers to how the dictators of that time, by having exclusive control of the means of mass communication, as well as by shamelessly deploying dehumanizing and Orwellian language, were able to change the ‘names’ of races, ethnicities, and previously prohibited forms of governmental behavior in ways that seemed to reverse age old human values wholesale and to reverse the moral dimensions of the rule of law that depended on them. Today, not just the dictatorial demagog but virtually any random individual or hate group has the capacity to even more massively and rapidly spread hate speech and misinformation simply by having access to the Internet. We suggest that what Milosz termed a ‘reversal’ of ‘values’, is, in bioscientific terms, a reversal of ‘empathy’.

We will now return to reviewing the scientific evidence which suggests that pain empathy is the source of such ‘values’ or that it is the *emotional enforcement mechanism* of what are variously termed, ‘universal-’, ‘human-’, or ‘equality-based-’, ‘-rights’.

### Adding Science to the Law and Philosophy

In recent years, neuroscientific evidence has been recruited to support legal theory on the role of empathy and pain in law. Most of this ‘neurolaw’ scholarship has applied neuroscience to criminal law and punishment theory. For instance, the discovery that sociopaths or criminals may have *impaired* neural empathic mechanisms has raised the question about how this might bear on traditional notions of criminal intent or *mens rea*.^79^

A few other scholars, including the authors of this paper, have instead drawn upon the neuroscience of empathy to illuminate how neural empathic mechanisms motivate rights-based conduct in the *healthy brain* or supply the neural foundation for the moral dimensions of law or equality-based rights.^80^ Professor James Duffy has hypothesized that ‘the emergence of empathy along with emotions such as shame and guilt provided the evolutionary impulse to promote the development of more egalitarian social systems where decisions are not driven by fear but by loyalty and avoiding pain towards others’.^81^

In addition, some scholars, including the authors of this paper, have investigated the application of the *neuroscience of pain* to the moral dimensions of law.^82^ Professor Amanda Pustilnik has argued against hedonic, subjectivist views that—because pain can now be increasingly ascertained to some degree by neuroimaging—neuroscientific data or physiological measurements of actual pain may eventually replace legal theory, concepts, or ‘values’ regarding the theoretical relation of pain to legal rights or duties.^83^

Pustilnik, besides arguing that data from the neuroimaging of pain does not admit of precise interpretation, argued that pain provides a basis for the moral dimensions of law not just because it exists as a scientifically discoverable, measurable physical entity—that can thus reasonably be attributed to others and the serious gratuitous infliction of which law should seek to detect, measure, and prevent—but due to pain's function as a *heuristic* in related areas of legal and moral theory:

In these areas, pain serves as a heuristic to reflect concerns about the *categories of actors* who deserve *empathy* and protection. Concern about pain reflects who (and what) we understand as being sufficiently *like us* to morally mandate protection from certain degrees of physical suffering.^84^

Pustilnik's words compare to the thesis in this paper, ie that the degree to which others are perceived as like us or human plays a role in the degree to which we empathize with and wish to alleviate their pain. We posit that this implicit sense of whether others are sufficiently like us or ‘human’ determines whether they automatically activate, in us, the neural affective components of pain that create what is commonly called, ‘empathy’, and that moves us to engage in rights-based decision-making and behavior toward them.

While Pustilnik focused more on the neuroimaging of the neural components of acute, *physical* or *nocioceptive*, pain and only touched briefly on the neural affective components of pain,^85^ she concluded that ‘[I]t is through the suffering of self that we understand the wrongfulness of causing gratuitous suffering to others; [and that] some of this is direct, *empathic*, and *likely physiological’*.^86^ However, she did not go on to address the findings by Singer et al.^87^ suggesting that the neural affective components of pain are the source of the empathy to which she refers and she did not address new theory that pain is also a *homeostatic emotion* involved in interoception as well as nocioception.^88^

This new view of pain as a homeostatic emotion is important in supporting legal theory that pain, in various forms, motivates legal compliance. From a biological versus philosophical view, the term, ‘motivation’, connotes *emotional* motivation. Pain has long been a mystery, for, like emotion, and it appeared anecdotally to have a strong motivational component. Nevertheless, while emotion is by definition, both a *feeling* and a *motivation*, previous scientific evidence and theory led to the view that pain was simply a sensation or *feeling*. However, recent neuroscientific evidence does suggest that pain has a *neural motivational or behavioral* component as well. This has led to an influential new theory, by neuroscientist, A.D. ‘Bud’ Craig, that pain is in fact a homeostatic *emotion* with behavioral motivational power.^89^

In practical terms, pain is thus not just a painful feeling in response to a dangerous or noxious stimuli, but a concomitant motivation to ‘act’ in accord, or to flee, avoid, shun, or attack the stimuli that evoke such feelings to ensure survival or homeostasis, whether the stimulus is a poisonous plant, snake, insect or any other non-human or human source of pain.

Craig points out, that while previous theory viewed pain as simply an unpleasant feeling, recent *f*MRI studies, like that of Singer et al., reflect that not only the AI or ‘limbic sensory cortex’ involved in creating a *feeling*-based cortical imaging of the body are activated in pain, but the ACC or ‘limbic *motor* cortex’ involved in *behavioral motivation* is also activated.^90^ Craig's theory is based not only upon neuroimaging studies in humans but also on functional anatomical findings in lower animals.^91^

To elaborate on the function of homeostatic emotions, they create a feelings-based sense of the homeostatic boundaries or state of one's body that virtually distinguishes self from non-self and further emotionally motivates behavior that enhances homeostatic integrity or survival. Craig has described this as an ‘afferent neural system in [nonhuman] primates and in humans that represents all aspects of the physiological condition of the physical body’ and as a ‘system [that] constitutes a representation of “the material me”, and [that] might provide a foundation for subjective feelings, emotion and self-awareness’.^92^

Singer's team, which cited Craig's theory as consistent with their data,^93^ found that the neural affective components of pain provide, beyond just a feeling-based sense of awareness of self, a sense of the feelings or pain of others as projected onto the self by neural internal simulation, or a brain-simulated empathic sense of ‘self as other’ that breaches the sense of physical separation between the ‘material me’ and other humans.^94^

Based upon this, we have hypothesized, in a previous paper, that pain empathy is the neural basis of what has been referred to anecdotally, as well as by legal, political, and moral theorists, as the ‘“sense’ of equality’.^95^ For the neural mechanisms of pain empathy make one ‘feel’ virtually ‘equal to’ or ‘one with’ others by creating a brain-simulated sense of ‘self as other’, whereby one automatically feels such others’ pain or its emotional components, anecdotally experienced as the ‘pangs’ of sympathy and compassion, which have strong, prosocial, emotive force.

This ingenious neurobiological mechanism essentially ‘tricks’ one's own body into ‘feeling’ as if its homeostatic boundaries or ‘material me’ have been expanded to incorporate other conspecifics. This, in turn, automatically, emotionally *motivates* one to act in the survival interests of such others, because their survival interests *feel* like one's own and their pain and suffering virtually become one's own through *neural simulation*.

There are many types or, what researchers call, ‘domains’, of empathy. However, one of the reasons that we proposed that it is *pain* empathy that provides the neural basis of the sense of equality, and that motivates behavior in accord with equality-based rights, is because the study by Singer et al., found that the subject did not have to *directly observe* another person in pain, but only be given an arbitrary ‘cue’ or *sign* that another person was in pain in order to exhibit neural empathic pain responses.^96^ Further, as noted by Singer's team, this triggering by cue distinguishes pain empathy from other forms of embodied empathy, such as emotional contagion, which require the *direct observation* of a conspecific exhibiting overt signs of an emotion, eg fear, in order for the observing animal to feel or ‘catch’ the same emotion.^97^ This would suggest that pain empathy can be felt for unseen, unknown humans in the abstract, which would be necessary, if it were to provide the emotional motivation to agree, and act in accord, with *universal* human rights.

The other reason that we propose that *pain* empathy is the neural foundation of the sense of equality long believed to motivate rights-based attitudes and behavior, is because evidence from clinical psychiatry and emerging findings in neuropsychiatry suggest that it is a *lack* of *pain empathy* that leads to pathologic antisocial attitudes, conduct, and violence. We will refer to a variety of diagnostic terms for, and categories of antisocial behavior, including ‘conduct disorder’, ‘antisocial personality disorder (APD)’ ‘sociopathy’, and ‘psychopathy’, and we may use the latter two terms interchangeably in general reference.

The American Psychiatric Association's *Diagnostic and Statistical Manual of Mental Disorders V* (*DSM-V*) defines antisocial personality disorder (APD) under the diagnostic code 301.7 and describes some of its diagnostic features thus: ‘The essential feature of antisocial personality disorder is a pervasive pattern of disregard for, and violation of, the rights of others that begins in childhood or early adolescence and continues into adulthood’.^98^

The *DSM-V* describes other associated features that support the diagnosis as follows: ‘Individuals with antisocial personality disorder frequently lack empathy and tend to be callous, cynical, and contemptuous of the feelings, rights, and sufferings of others’.^99^ ‘Suffering’ is *affective* or *emotional* pain, and the pain study above by Singer et al. (2004) revealed that empathy for the pain and suffering of others is an embodied empathy involving activation of the neural affective components of pain in which the ACC and bilateral AI appear to figure prominently.

Neuroscientific research suggests that APD, conduct disorder, and criminal behavior may be linked to deficiencies in this embodied empathy for pain and suffering or in differences in the volume or activity of these brain areas involved in empathy.

For example, studies of adolescents with conduct disorder, a precursor to APD, have shown reduced gray -matter volume in the AI, which correlated with observed levels of empathy and aggressive behavior.^100^ Studies of subjects with APD have revealed reduced activity in the AI and amygdala, which could reflect deficits in embodied empathy and processing of emotion.^101^ In addition, there is also significant evidence of differences between the brains of psychopaths or sociopaths and normal controls in paralimbic structures in the brain involved in emotion and social reactions.^102^ A recently published *f*MRI study found that adult criminals with low levels of anterior ACC activity were twice as likely to be rearrested within four years of release than offenders with high activity in this area.^103^

There is non-human, primate research from the 1940's that provides impressive neuroscientific evidence that pain empathy is vital to prosocial behavior. Wilbur Smith and Arthur Ward studied chimps after the animals had undergone a cingulotomy or removal of the brain area, which is a homolog to the human ACC that is implicated in pain empathy in people, and these animals appeared to lose the capacity to interact socially or to treat others right.^104^ Ward wrote that such an animal:

shows no … acts of affection towards its companions. In fact, it treats them as it treats inanimate objects and will walk on them, bump into them if they happen to be in the way, and will even sit on them …. It acts under all circumstances as though it had lost its social conscience.^105^

An interesting aspect of this study is that, not only did these animals appear to lose the ability to care about how they treated their conspecifics (ie members of the animals’ own species), this seemed to be further associated with a failure to even *recognize their conspecifics* as such.^106^ This points to the next section in which we examine whether the failure of a human subject's brain to implicitly associate a conspecific with its own species could automatically inhibit pain empathy for that conspecific.

If it is an underlying failure of the neural mechanisms of pain empathy that results in lack of rights-based attitudes and behavior or in antisocial prejudice and violence, then how—based on what is known about these mechanisms—might *dehumanization* reduce pain empathy in large numbers of normal, healthy people who are not sociopaths, or how might it cause a failure of healthy neural empathic pain mechanisms to respond to the victims of such dehumanization?

In practical terms, how might implicit dehumanization take neural empathic pain mechanisms offline, similar to the way they appear to fail to function in sociopathic humans with apparent physical deficits in the same, or in chimps post-cingulotomy, yet with the difference being that such mechanisms only fail to respond to the targets or victims of such dehumanization, while leaving empathy for *other conspecifics* intact?

## REQUIREMENT OF A CONSPECIFIC OR ‘HUMAN’ TO EVOKE EMPATHY?

We can provide, in the scope of this chapter, only a brief, simplified review of the limited neuroscientific findings in human and non-human primates which thus suggest that neural internal simulation mechanisms, such as those that appear to be involved in empathy, are best activated by conspecifics or individuals that are associated, in the empathizing animal's brain, with its own species or with traits associated with its species.

First, it is important to note that such simulation or mirroring mechanisms appear to operate automatically, without conscious awareness or control. This suggests that, if such mechanisms respond to conspecifics, the determination, of whether a human target represents a conspecific, would not be based on whether the subject consciously viewed the target as human but on whether the target exhibited those traits that had become most strongly, *implicitly* associated with the neural representation of ‘human’ in the subject's brain. This non-conscious nature of mirror systems has been described as follows:

this ‘mirroring capacity’ of the brain originates at a much deeper level than the level of phenomenal consciousness. The ‘mirroring’ can be enacted not only completely unconsciously, but is also coded at quite a low level of brain functioning—at the microscale of its neural performance. The mirror neurons become activated independently of the agent of the action —the self or the third person whose action is observed.^107^

Let us now move on to look at the evidence that the subject mirror neural simulation mechanisms appear to be more responsive to a subject's *conspecifics*.

In a study using *f*MRI done by Buccino et al. to test the neural circuits that appear to be involved in the recognition of motor actions performed by non-conspecifics, results indicated that the human mirror system is more active when the motor actions of *other humans* are observed, than those of *other species*, in this case, monkeys and dogs.^108^ It is interesting that these species are two of those most commonly used to refer to social groups that the hate speaker wishes to dehumanize.

Not only did this study suggest the human mirror system responds more actively to humans than non-conspecifics, it suggested that when the human mirror system does respond to the actions of a non-conspecific, it appears to be only when the other species exhibits traits associated with the human species, for example, when the action of the non-conspecific is also part of the human motor repertoire.^109^

The human mirror system failed to activate when the actions observed were those that are only exhibited by other, non-human species.^110^ There is also evidence that the human mirror system is more readily activated when the observer and observed are similar to one another, not just in the sense that both are human or performing acts associated with humans, but when, for example, both are dancers.^111^

In studies of mirror neurons in monkeys, the monkey's mirror neurons were activated both when the monkey grasped a piece of food and when the animal passively observed a human experimenter grasp the food.^112^ Thus, the monkey's mirror neurons fired in response to the actions of a different but closely related species, humans, who exhibit a motor repertoire of grasping food with the hands that is similar to that of the monkey.

There is also evidence that mirror neurons respond generally only to living or biological entities versus objects. For example, in the early studies of monkeys, the animal's mirror neurons failed to fire upon mere presentation of the object to be grasped.^113^ The study also showed that mirror neurons in the monkeys were not activated by observation of the grasping task when performed with a tool, artificial mechanical grasping device or pliers, rather than with the hand.^114^ This suggests that the goal-oriented motor task of grasping a piece of food might fail to activate mirror neurons if it is performed or associated with a non-biological entity, object, or instrument.

A later study in monkeys did reveal a population of mirror neurons that responded to some actions involving the use of tools.^115^ Yet, another study of humans showed that, though observation of both a human and robotic hand could facilitate automatic imitation (simulation) in humans, that even when the human and robotic hands looked alike, the human hand had a more robust effect upon performance.^116^

Though most of the above cited research was performed to investigate the mirroring of motor *actions* and *action understanding* or to study such mirroring's relation to attributing states of mind, intentions, or goals to the actor, and we are here instead examining pain empathy or the mirroring of others’ *pain*, the above evidence that pain empathy also involves a mirroring system or mirror neurons supports a tentative hypothesis that the neural mechanisms of pain empathy may also be less responsive to social targets who are not conspecifics or not like the self, or who are implicitly associated with *subhuman* life and traits or with *non-living* objects, tools, and instruments. This would comport with anecdotal, as well as philosophical, observations that people who are dehumanized, instrumentalized, or objectified are treated in ways that suggest a lack of empathy for their pain and suffering as well as their basic rights.

## CONCLUSION

If implicit dehumanization of a target social group dampens the response of neural empathic pain mechanisms to that group, this could help shed light on the long anecdotal and historical relation between dehumanization and the catastrophic human rights abuses, such as torture and genocide, that have stubbornly persisted, along with implicit biases, across time and legal culture. It could also shed light upon the insidiously *unconscious* nature of social prejudice and the impact of dehumanizing hate speech and current legal fictions of ‘personhood’ in enabling it. One bioethics commentator recently described the growing impact of neuroscience and related technology on human rights.^117^ Perhaps, neuroscience will place the rights to free speech and social equality into sharper conflict. Perhaps, it will refute the folk psychology that name calling is harmless or put to bed the nursery rhyme, ‘Sticks and stones will break my bones/But words will never harm me’. For, as emerging neuroscience, children bullied online and Holocaust survivors equated with cattle can tell you, such platitudes may not be true, even in a literal, physical sense.

Finally, though we hypothesize that dehumanization dampens pain empathy for dehumanized individuals and groups and thereby puts their human rights at risk, we do not contend that our hypothesis, as such and alone, is a sufficient basis for specific policy recommendations regarding whether various legal restrictions on dehumanizing or ‘hate’ speech would be effective in reducing implicit biases and associated human rights abuses. Perhaps, education—or becoming more conscious of the non-conscious neural mechanisms of empathy and moral restraint and the possible effect of our words upon them—is a start.

## Footnotes

1 In proposing a scientific hypothesis to explain and emphasize the potential harms of dehumanization, we were acutely aware of the irony that one of the scientific terms, in the research we've cited, refers to human research subjects as ‘social targets’ or ‘targets’, a term also used historically to objectify or dehumanize chosen groups. Some bioethicists criticize the use of objectifying terms, such as ‘target’ and ‘subject’, in clinical research. Regardless of the insensitivity of these word choices in science, such terms and associated concepts are deeply embedded in the social- and neuroscience of empathy. For readers not familiar with the term, ‘social target’, as it is used in social scientific research, the following is a simplified definition: a ‘target’ is an individual (human or animal) in response to whom/which another individual of the same species may show a social behavior or emotion. For example, if one human observes another human ‘social target’ (a potential *recipient* of a behavioral, linguistic, or emotional response from the observer) and that social target appears to be in pain, the observer may (or may not) exhibit empathy for the ‘target's’ pain. Though we avoid unnecessary use of the *noun* ‘target’ to refer to humans, we still use at times in this paper for scientific purposes. When we do, we have tried to add language to help minimize any objectifying implications, by, for example, referring to social targets as ‘*human* social targets’ or ‘*dehumanized* targets’ (as only humans can be dehumanized). This is not to be confused with use of the term ‘target’ as a *verb* often used to refer to the behavior or speech acts of hate speakers who intentionally, explicitly dehumanize and actively ‘target’ select groups with vitriol, discrimination, and violence.

2 See Nick Haslam, *Dehumanization: An Integrative Review*, 10 Pers. Soc. Psychol. Rev. 252 (2006).

3 See Kimberly Costello & Gordon Hodson, *Exploring the Roots of Dehumanization: The Role of the Animal-human Similarity in Promoting Immigrant Humanization*, in 13 Group Process and Intergroup Relations 3, 22 (2010).

4 See eg Naomi Struch & Shalom H. Schwartz, *Intergroup Aggression: Its Predictors and Distinctness from In-Group Bias*, 56 J. Pers. Soc. Psychol. 364 (1989).

5 Haslam, *supra* note 1.

6 Gordon W. Allport, The Nature of Prejudice (25th anniversary ed. 1979).

7 Brian Mullen & Tirza Leader, *Linguistic Factors: Antilocutions, Ethnonyms, Ethnophaulisms, and Other Varieties of Hate Speech*, in On the Nature of Prejudice: Fifty Years After Allport 192 (John Dovidio et al. eds, 2005).

8 Allport, *supra* note 6, at 48, 67, 178–87.

9 Allport, *supra* note 6, at 50.

10 See Hannah Arendt, Eichmann in Jerusalem: A Report on the Banality of Evil (Penguin Books 2006) (1963).

11 Please note that, though we propose a neural explanation for social prejudice and related violence, we acknowledge that these neural mechanisms are only one potential factor contributing to such social attitudes and behavior. Other factors obviously include the social environment, ideology, cultural beliefs and myths, and linguistic practices, the latter two of which our hypothesis suggests might impact the function of subject neural mechanisms. While we focus more on evidence from neuroscience, because we are presenting a neurological hypothesis, we do not wish to imply that the wealth of evidence and analysis from other fields should be discounted in favor of neuroscientific data or methodologies. For neuroscience is just beginning to be able to investigate fine-grained aspects of the mind and social behavior that scholars in other disciplines have studied for considerably longer. Social neuroscience, at its best, is guided, in formulating hypotheses and interpreting findings, by insights from other fields, such as social science, psychology, philosophy, law, feminist studies, race theory, arts, history, and other humanities, each of which still provides more insight into the human mind than neuroimaging or single-neuron recordings.

12 Lasana T. Harris & Susan T. Fiske, *Perceiving Humanity or Not: A Social Neuroscience Approach to Dehumanized Perception*, in Social Neuroscience: Toward Understanding the Underpinnings of the Social Mind 123, 130–31 (Alexander Todorov et al. eds, 2011) (footnote omitted).

13 A large body of literature details the scientific evidence of implicit bias. For two reviews aimed at a legal audience, see Anthony G. Greenwald & Linda H. Krieger, *Implicit Bias: Scientific Foundations*, 94 Cal. L. Rev. 945 (2006); Kristin A. Lane, Jerry Kang & Mahzarin R. Banaji, *Implicit Social Cognition and Law*, 3 Ann. Rev. L. Soc. Sci. 427 (2007). See generally Cheryl Staats & Charles Patton, Kirwan Institute for the Study of Race and Ethnicity, State of the Science: Implicit Bias Review 2013 (2013), http://kirwaninstitute.osu.edu/docs/SOTS-Implicit_Bias.pdf (last accessed May 6, 2015).

14 See generally Greenwald & Hamilton Krieger, *supra* note 13.

15 Id.

16 Id.

17 Staats & Patton, *supra* note 13.

18 See Michael Billig, *Henri Tajfel's ‘cognitive aspects of prejudice’ and the Psychology of Bigotry*, 41 Brit. J Soc. Psychol. 171, 188 (2002); Christian Tileagă, *Ideologies of Moral Exclusion: A Critical Discursive Reframing of Depersonalization, Delegitimization and Dehumanization*, 46 Brit. J. Soc. Psychol. 717, 737 (2007).

19 Harris & Fiske, *supra* note 12.

20 Mina Cikara, Jennifer Eberhardt & Susan Fiske, *From Agents to Objects: Sexist Attitudes and Neural Responses to Sexualized Targets*, 10 J. Cogn. Neurosci. 1, 12 (2010).

21 Id.

22 Lasana Harris & Susan Fiske, *Dehumanizing the Lowest of the Low: Neuroimaging Responses to Extreme Out-Groups*, 17 Psychol. Sci. 847–53 (2006).

23 See eg Nathan A. Heflick & Jamie L. Goldenberg, *Dehumanization: A Threat and Solution to Terror Management*, in Humanness and Dehumanization 111,126 (Paul G. Bain et al. eds, 2014); Albert Bandura, *Selective Moral Disengagement in the Exercise of Moral Agency*, 31 J. Moral Educ., 101, 119 (2002).

24 See eg Jacques-Philippe Leyens, *Humanity Forever in Medical Dehunamization*, in Humanness and Dehumanization 167,185 (Paul G. Bain et al. eds, 2014) (providing a review of research and theory on medical dehumanization).

25 See generally Joseph Sandler, *The Concept of Projective Identification*, in 10 Bulletin of the Anna Freud Centre 33, 49 (1987).

26 Eg Spike Peterson & Laura Parisi, *Are Women Human? It's not an Academic Question*, in Human Rights Fifty Years On: A Reappraisal 132, 160 (Tony Evans ed., 1998).

27 Stephanie D. Preston & Frans B. M. de Waal, *Empathy: Its Ultimate and Proximate Bases*, 25, Behav. Brain Sci. 1, 72 (2002). Please note that the hypothesis we present in this paper, though consistent with PAM, is a much narrower theory that relies on a smaller range of data. We cite PAM not only because it is generally consistent with our hypothesis, but to offer readers a reliable, single source of background material on the science and related theories of empathy. In addition, this particular journal article by Preston and de Waal has a separate section at the end with open peer commentary on, and critiques of, their model.

28 *Id.* at 2, 5.

29 *Id.* at 4.

30 Vittorio Gallese et al., *Action Recognition in the Premotor Cortex*, 119 Brain 593 (1996); Giacomo Rizzolatti et al., *Premotor Cortex and the Recognition of Motor Actions*, 3 Cogn. Brain Res. 131 (1996).

31 Gallese, et al., *supra* note 30; Rizzolatti, et al., *supra* note 30.

32 Id.

33 Id.

34 See Giacomo Rizzolati & Laila Craighero, *The Mirror-Neuron System*, 27 Annu. Rev. Neurosci. 169 (2004) (providing an overview of this evidence of a mirror-neuron system).

35 See Luigi Cattaneo & Giacomo Rizzolati, *The Mirror Neuron System*, 66 Arch. Neurol. 557 (2009) (providing a brief review of research regarding the mirror-neuron system).

36 Tania Singer et al., *Empathy for Pain Involves the Affective but not Sensory Components of Pain*, 303 Science 1157, 1162 (2004).

37 Id.

38 Id.

39 Id.

40 *Id.* at 1158.

41 William D. Hutchison et al., *Pain-related Neurons in the Human Cingulate Cortex*, 2 Nature Neurosci. 403 (1999).

42 Id.

43 Id.

44 Id.

45 India Morrison & Paul Downing, *Organization of Felt and Seen Pain Responses in Anterior Cingulate Cortex*, 37 Neuroimage 642 (2007).

46 Id.

47 But cf. Gregory Hickok, The Myth of Mirror Neurons: The Real Science of Communication and Cognition (2014) (Provides a recent, in depth, critical analysis of mirror neuron theory as well as the methods and interpretations of data in the initial studies in Italy in which mirror neurons were discovered).

48 See generally Tania Singer & Claus Lamm, *The Social Neuroscience of Empathy*, 1156 Ann. N Y Acad. Sci. 81 (2009) (providing an accessible review of the advancement of the social neuroscience of empathy since the earlier, pioneering work published by Singer et al. in 2004).

49 See Cesare Beccaria, On Crimes and Punishments 10, 13 (Henry Paolucci trans., Prentice Hall 1963) (1764); Jeremy Bentham, The Principles of Morals and Legislation 29, 42 (Prometheus Books 1988) (condensed version of Bentham's 1781 work, An Introduction to the Principles of Morals and Legislation).

50 Bentham, *supra* note 49, at 40.

51 See eg Dred Scott v. Sanford, 60 U.S. 393 (1857); Citizens United v. F.E.C., 558 U.S. 310 (2010).

52 Billig and Tileagă, *supra* note 18.

53 Sophie Oliver, *Dehumanization: Perceiving the Body as (In)Human*, in Humiliation, Degradation, Dehumanization 85, 97 (Paulus Kaufmann et al. eds, 2011).

54 *Id*. at 95, 96.

55 Our proposal should not be confused with the so-called ‘*human* personhood’ movement that endeavors to secure, for the human ovum, embryo, and pre-viable fetus, legal personality, personhood, or, in effect, the same legal status and rights as live, post-birth humans. In fact, this technical contradiction in terms, ie ‘human personhood’, is an example of the confusion we seek to avoid by our suggestion that one's entitlement to human rights should be based on whether one is ‘human’, rather than on whether one is anecdotally or legally labeled a ‘person’. This practice may help to avoid the confusion, in the law and the public mind, regarding the squishy concept of personhood, confusion which terms like, ‘human personhood’, reinforce and exploit. Perhaps, we should at last correct Descartes’ error, in *law*, as it has been corrected in science, by determining the human spirit inseparable from the human body and brain?

56 George Lakoff & Mark Turner, More than Cool Reason: A Field Guide to Poetic Metaphor 60, 65 (University of Chicago Press 1989).

57 PETA Deutschland v. Germany, no. 43481/09, ECHR 2012.

58 *Id*. at ¶ 44.

59 See Citizens United v. F.E.C., 558 U. S. 310, 393–479 (2010) (Stevens, J., dissenting).

60 *Id.* at 428.

61 See case cited *supra* note 59 at 469 (citing Austin v. Michigan Chamber of Commerce, 496 U.S. 652 at 660 (1990)).

62 *Id.* at 470.

63 *Id.* at 475.

64 *Id.* at 447, 463.

65 *Id.* at 466.

66 Brandenburg v. Ohio, 395 U.S. 444, 444 (1969).

67 Alexander Tsesis, *Dignity and Speech: The Regulation of Hate Speech in a Democracy*, 44 Wake Forest L. Rev. 497, 502 (2009).

68 See Jeremy Waldron, The Harm in Hate Speech 11, 17 (2012); Steven Heyman, *Hate Speech, Public Discourse, and the First Amendment*, in Extreme Speech and Democracy 158, 181 (Ivan Hare & James Weinstein eds, 2009).

69 Robert Mark Simpson, *Dignity, Harm, and Hate Speech*, 32 L. & Philos. 701, 704 (2013).

70 S. 2219, 113th Cong. (2013–2014).

71 H.R. 3873, 113th Cong. (2014).

72 Tiffany Kamasara, *Planting the Seeds of Hatred: Why Imminence Should No Longer Be Required to Impose Liability on Internet Communications*, 29 Cap. U. L. Rev. 835, 837 (2002).

73 See Sarah Sorial, *Hate Speech and Distorted Communication Rethinking the Limits of Incitement*, 34 L.& Phil. 299–324 (2015); John P. Cronan, *The Next Challenge for the First Amendment: The Framework for an Internet Incitement Standard*, 51 Cath. U. L. Rev. 425 (2002).

74 Whitney v. California, 274 U.S. 357, 377 (1927).

75 With regard to this proposition concerning increased use of passively dehumanizing, vilifying, rhetoric in US political discourse and reporting, we depart from the rigorous practice in legal, academic writing of providing citation to specific examples in support and instead ask readers to draw upon their own, personal experience and observation for evidence regarding the veracity of this general proposition. While we make this general proposition that such rhetoric is currently a widespread, cultural, linguistic practice utilized by diverse ideologies, politicians, journalists, academics, pundits, and comics (and in mainly well-intentioned ways), and, though we will also offer only *general* versus specific descriptions of this form of rhetoric, we refrain from providing specific examples or naming names, so as to avoid the appearance of engaging in only more political name calling or of giving further currency to any terms that dehumanize or demean.

76 We adhere to the same practice here, as outlined in *supra* not*e* 75, of refraining from pointing to or accusing specific individuals, organizations, or groups, or from providing sample quotes.

77 Sorial, *supra* note 73, abstract.

78 Czeslaw Milosz, *Ruins and Poetry*, The New York Review of Books, Mar. 17, 1983, at 2.

79 For an edited collection of works that focuses on various implications of neuroscience for criminal responsibility, see *infra* note 81.

80 See Gail B. Murrow & Richard W. Murrow, *A Biosemiotic Body of Law: The Neurobiology of Justice*, 26 Int'l J. Sémiotics L 275 (2012).

81 James D. Duffy, *What Hobbes Left Out: The Neuroscience of Compassion and its Implications for a New Common-wealth*, in 13 Law and Neuroscience: Current Legal Issues 433–436 (Michael Freeman ed., 2011).

82 Murrow & Murrow, *supra* note 80, at 298, 300.

83 Amanda C. Pustilnik, *Pain as Fact and Heuristic: How Pain Neuroimaging Illuminates Moral Dimensions of Law* 97 Cornell L. Rev. 801–847 (2012).

84 *Id.* at 806 (emphasis added).

85 *Id.* at 812.

86 *Id.* at 847 (emphasis added).

87 Singer, *supra* note 36.

88 A.D. Craig, *A New View of Pain as a Homeostatic Emotion*, 26 Trends Neurosci. 303 (2003).

89 *Id*.

90 A.D. Craig, *How Do You Feel? Interoception: The Sense of the Physiological Condition of the Body*, 3 Nat. Rev. Neuorsci. 655, 666 (2002).

91 See eg A.D. Craig & David Andrew, *Responses of Spinothalamic Lamina I Neurons to Repeated Brief Contact with Heat Stimulation in the Cat*, 87 J. Neurophysiol. 1902 (2002).

92 Craig, *supra* note 88, at 663 (footnotes omitted).

93 Singer et al., *supra* note 36, at 1160, 1161.

94 *Id.* at 1158.

95 Murrow & Murrow, *supra* note 80, at 298, 300.

96 Singer et al., *supra* note 36, at 1158.

97 *Id*.

98 American Psychiatric Association, Diagnostic and Statistical Manual of Mental Disorders, (5th ed. 2013).

99 Id.

100 See Philipp Sterzer et al., *A Structural Neural Deficit in Adolescents with Conduct Disorder and its Association with Lack of Empathy*, 37 Neuroimage 335 (2007).

101 See Niels Birbaumer et al., *Deficient Fear Conditioning in Psychopathy: A Functional Magnetic Resonance Imaging Study*, 62 Arch. Gen. Psychiatry 799 (2005).

102 See Kent A. Kiehl, *A Cognitive Neuroscience Perspective on Psychopathy: Evidence for Paralimbic System Dysfunction*, 142 Psychiatry Res, 107 (2006).

103 See Eyal Aharoni et al., *Neuroprediction of Future Rearrest*, 110 Proc. Natl. Acad. Sci. 6223 (2013).

104 See Wilbur K. Smith, *The Functional Significance of the Rostral Cingular Cortex as Revealed by its Response to Electrical Stimulation*, 8 J. Neurophysiol. 241 (1945); Arthur A. Ward, *The Anterior Cingulate Gyrus and Personality*, 27 Res. Publ. Assoc. Nerv. Ment. Dis. 438 (1948).

105 Ward, *supra* note 104, at 438, 445.

106 Id.

107 Maxim Stamenov & Vittorio Gallese, eds, *Introduction* to *Mirror Neurons and the Evolution of Brain and Language*, in 42 Advances in Consciousness Research 2 (2002).

108 See Giovanni Buccino et al., *Neural Circuits Involved in the Recognition of Actions Performed by Nonconspecifics: An* f*MRI study*, 16 J. Cogn. Neurosci.114 (2004).

109 Id.

110 Id.

111 See Beatriz Calvo-Merino et al., *Action Observation and Acquired Motor Skills: An* f*MRI Study with Expert Dancers*, 15 Cereb. Cortex 1243 (2005).

112 Gallese et al., *supra* note 30.

113 Id.

114 Id.

115 See Pier F. Ferrari, Stefano Rozzi & Leonardo Fogassi , *Mirror Neurons Responding to Observation of Actions Made with Tools in Monkey Ventral Premotor Cortex*, 17 J. Cogn. Neurosci. 212 (2005).

116 See Clare Press et al., *Robotic Movement Elicits Automatic Imitation*, 25 Cogn. Brain Res. 632 (2005).

117 Mark S. Frankel, *Considering Advances in Neuroscience Through the Lenses of Law and Human Rights*, 1 J. L. & Biosci 215, 217 (2014).

